# Pre‐analytical factors affecting the establishment of a single tube assay for multiparameter liquid biopsy detection in melanoma patients

**DOI:** 10.1002/1878-0261.12669

**Published:** 2020-04-04

**Authors:** Svenja Schneegans, Lelia Lück, Katharina Besler, Leonie Bluhm, Julia‐Christina Stadler, Janina Staub, Rüdiger Greinert, Beate Volkmer, Mikael Kubista, Christoffer Gebhardt, Alexander Sartori, Darryl Irwin, Elina Serkkola, Taija af Hällström, Evi Lianidou, Markus Sprenger‐Haussels, Melanie Hussong, Peter Mohr, Stefan W. Schneider, Jonathan Shaffer, Klaus Pantel, Harriet Wikman

**Affiliations:** ^1^ Department of Tumor Biology University Medical Center Hamburg‐Eppendorf Germany; ^2^ Centre of Dermatology Elbe Clinics Buxtehude Germany; ^3^ Department of Dermatology and Venereology University Medical Center Hamburg‐Eppendorf Germany; ^4^ TATAA Biocenter AB Gothenburg Sweden; ^5^ Department of Gene Expression Institute of Biotechnology Czech Academy of Sciences Vestec Czech Republic; ^6^ Agena Bioscience GmbH Hamburg Germany; ^7^ Orion Pharma Orion Corporation Espoo Finland; ^8^ Analysis of Circulating Tumor Cells Lab of Analytical Chemistry Department of Chemistry University of Athens Greece; ^9^ QIAGEN Inc/GmbH Frederick MD USA; ^10^ QIAGEN Inc/GmbH Hilden Germany; ^11^ Present address: AstraZeneca Espoo Finland; ^12^ Present address: Institute for Molecular Medicine Finland University of Helsinki Finland

**Keywords:** CTC, ctDNA, EV, liquid biopsy, melanoma, miRNA

## Abstract

The combination of liquid biomarkers from a single blood tube can provide more comprehensive information on tumor development and progression in cancer patients compared to single analysis. Here, we evaluated whether a combined analysis of circulating tumor cells (CTCs), circulating tumor DNA (ctDNA), and circulating cell‐free microRNA (miRNA) in total plasma and extracellular vesicles (EV) from the same blood sample is feasible and how the results are influenced by the choice of different blood tubes. Peripheral blood from 20 stage IV melanoma patients and five healthy donors (HD) was collected in EDTA, Streck, and Transfix tubes. Peripheral blood mononuclear cell fraction was used for CTC analysis, whereas plasma and EV fractions were used for ctDNA mutation and miRNA analysis. Mutations in cell‐free circulating DNA were detected in 67% of patients, with no significant difference between the tubes. CTC was detected in only EDTA blood and only in 15% of patients. miRNA NGS (next‐generation sequencing) results were highly influenced by the collection tubes and could only be performed from EDTA and Streck tubes due to hemolysis in Transfix tubes. No overlap of significantly differentially expressed miRNA (patients versus HD) could be found between the tubes in total plasma, whereas eight miRNA were commonly differentially regulated in the EV fraction. In summary, high‐quality CTCs, ctDNA, and miRNA data from a single blood tube can be obtained. However, the choice of blood collection tubes is a critical pre‐analytical variable.

## Graphical abstract

We assessed whether a combined analysis of circulating tumor cells, circulating tumor DNA, and microRNA in total plasma or extracellular vesicles from one blood sample is feasible. We show that high‐quality data from a single blood tube can be obtained in melanoma patients. However, the choice of the tube affects the outcome of the analysis, underlining the importance of pre‐analytical factors.

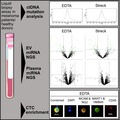

**Abbreviations**
cfDNA: cell‐free circulating DNA
CTC: circulating tumor cell
ctDNA: circulating tumor DNA
EM: electron microscopy
EV: extracellular vesicle
FC: fold change
HD: healthy donor
miRNA: MicroRNA
MM: metastatic melanoma
NGS: next generation sequencing
NTA: nanoparticle tracking analysis
PBMCs: peripheral blood mononuclear cells
UMI: Unique Molecular Index
VAF: variant allele frequency

## 1 Introduction

Liquid biopsy has become an important alternative tool for cancer diagnostics and includes the analysis of circulating tumor cells (CTCs), circulating tumor DNA (ctDNA), extracellular vesicles (EVs), and circulating cell‐free microRNA (miRNA) (Alix‐Panabieres and Pantel, 2016; Anfossi *et al.*, 2018; Bardelli and Pantel, 2017; Girotti *et al.*, 2015; Zavridou *et al.*, 2018). The amounts of both CTCs and ctDNA are often very low, and the half‐life time of both biomarkers is short (1–2 h) (Diehl *et al.*, 2008; Meng *et al.*, 2004). In contrast, miRNA last up to 24 h in the bloodstream due to their inclusion in EVs or binding to (lipo)proteins (Cheng *et al.*, 2014; Mitchell *et al.*, 2008; Vickers *et al.*, 2011). However, their normalization and natural variation depend on many factors, which can be a drawback (Schwarzenbach *et al.*, 2015). Therefore, the use of multiple biomarkers found in the blood of patients might give the most comprehensive information to be used for patient monitoring and disease control (Anfossi *et al.*, 2018).

Different studies have shown the superiority of specific blood collection tubes for specific liquid biopsy assays (Alidousty *et al.*, 2017; Bartak *et al.*, 2018; Nikolaev *et al.*, 2018), but a comprehensive analysis of the major liquid biopsy analytes in one single tube is, to the best of our knowledge, still lacking. Since the occurrence, concentration, and stability of these biomarkers are distinct, different analyses might require different blood collection tubes.

The incidence of melanomas has increased dramatically over the past years (Erdmann *et al.*, 2013). Although the introduction of targeted therapies (e.g., against BRAF) and, in particular, immunotherapies have significantly improved the survival rates of some patients, the overall 5‐year survival rate is still only 25%. Melanomas show an exceptional high mutation rate and tumor heterogeneity (Gray *et al.*, 2015). Therefore, we chose here melanoma as a tumor entity with a need of a multiparameter liquid biopsy approach. In this study, we report that reliable ctDNA, CTC, and miRNA analysis can be performed from blood samples collected in a single collection tube from melanoma patients. We also show the suitability of an next generation sequencing (NGS)‐based total and EV‐associated miRNA detection workflow in melanoma patients and used a specific melanoma‐associated ctDNA mutation panel.

## 2 Materials and methods

### 2.1 Sample collection

Peripheral blood was collected from 20 metastatic melanoma (MM; AJCC stage IV) patients with progressive disease. Blood from 11 women and nine men was collected (mean age: 74). Nineteen patients were diagnosed with cutaneous melanoma and one patient with uveal melanoma. The study was approved by the local ethical committee (PV5932), conducted according to the Declarations of Helsinki, and all participants gave their written consent. The samples were collected and analyzed in a blinded manner.

The experimental procedure and used samples are shown in Fig. S1. Blood was collected in parallel in three different blood collection tubes, that is, EDTA (BD Vacutainer, Franklin Lakes, NJ, US), Streck cell‐free circulating DNA (cfDNA) BCT® (Streck, La Vista, NE, US), and Transfix (Cytomark, Buckingham, UK; 7.5 mL each). EDTA was chosen due to its broad applicability, whereas Streck is commonly used in ctDNA analyses and we have recently shown the suitability of Transfix for CTC analysis (Koch *et al.*, 2020). Plasma removal without hemolysis was not possible from Transfix tubes, so these tubes were only used for CTC analyses. The EDTA and Streck tubes were processed within 2–5 h after blood collection, and the Transfix tubes were processed on the next day according to the manufacturer's recommendations. For the miRNA analyses, peripheral blood was collected in Streck and EDTA tubes from five healthy donors (HD) and processed identically with the patients' samples. The HD were sex but not age matched.

### 2.2 Plasma extraction

Blood from both EDTA and Streck tubes was first centrifuged at 500 ***g*** for 10 min in order to separate plasma from peripheral blood mononuclear cells (PBMCs).

The supernatant of the plasma fraction was divided over two tubes (1.5–2 mL per tube) and plasma from Streck tubes designated for ctDNA extraction was centrifuged at 6000 ***g*** for 10, while EDTA tubes were centrifuged at 2500 ***g*** for 10 min. These plasma fractions were stored at −80 °C until cfDNA extraction. The supernatant of the second plasma fractions designated for miRNA extraction (both EDTA and Streck) was centrifuged at 2500 ***g*** for 10 min, and their supernatants were centrifuged once more at 2500 ***g*** for 15 min before the supernatant was stored (−80 °C) for EV and miRNA isolation.

### 2.3 ctDNA isolation

The QIAamp MinElute ccfDNA Mini Kit (Qiagen, Hilden, Germany) was used to isolate cfDNA from 2 mL plasma according to the manufacturer's instructions, and samples were eluted in 25 μL.

The cfDNA concentration was measured using the Qubit Fluorometric Quantitation System (Thermo Fisher Scientific, Waltham, MA, US). Quality measurements were performed with TapeStation (High Sensitivity D5000 ScreenTape; Agilent Technologies, Santa Clara, CA, US). Statistical testing of differences in ctDNA concentrations was assessed using Wilcoxon signed‐rank test in R.

### 2.4 ctDNA mutation analysis

For the identification of 86 hot‐spot mutations in 13 genes (Table S1) from the isolated ctDNA, the UltraSEEK™ Melanoma Panel (Agena Bioscience, Hamburg, Germany) was used. The enriched products were transferred onto SpectroCHIP®‐96 Arrays and analyzed with matrix assisted laser desorption/ionization‐time of flight mass spectrometry‐based MassARRAY® System (Agena Bioscience) according to the manufacturer's instructions (Mosko *et al.*, 2016).

### 2.5 CTC capture and enrichment

The PBMC fraction was processed with the ClearBridge ClearCell® FX1 System (Biolidics Limited, Singapore, Singapore) using program no. 1 (default setting) according to the manufacturer's instructions. Briefly, the PBMC fraction was treated with red blood cell lysis buffer (Biolidics), and the pellet was resuspended in 4 mL of resuspension buffer before being placed in the ClearCell FX1 System with a CTC Chip™FR1. The solution from the output was then centrifuged (500 ***g***, 10 min), and all supernatant except for the last 200 μL was discarded. The cell pellet was resuspended in the remaining 200 μL and transferred to a slide and left for air‐drying. In one patient, the blood processing cassettes for EDTA and Streck were damaged and could not be processed, whereas in one additional patient the Streck blood was coagulated.

### 2.6 CTC spiking experiments

To analyze whether plasma removal from whole blood has an influence on CTC recoveries, EDTA (*n* = 4) and Streck HD blood (*n* = 4) were spiked with 50 H1975‐GFP‐positive cells. Plasma was removed and processed with the ClearCell FX1 system (see above). The pellet was transferred into a 96‐well plate and screened for GFP‐positive cells. Statistical testing of the influence of plasma removal was analyzed using Wilcoxon signed‐rank test in r (R Foundation for Statistical Computing, Vienna, Austria). The recovery rates after plasma removal combined with our optimized staining protocols (see below) were estimated using 100 SK‐MEL‐28 cells using both EDTA and Streck tubes (*n* = 2 each).

### 2.7 CTC staining

Two different staining protocols for CTCs were used in this study. In the first ten samples as well as CTC spiking experiments with SK‐MEL‐28 cells, the cells were fixed with 3.7% formaldehyde for 10 min, then a blocking buffer containing 1× PBS, 5% goat serum, 5% v/v FcR blocking reagent (Miltenyi Biotec, Bergisch Gladbach, Germany), and 0.3% Triton™ X‐100 (Sigma‐Aldrich, St. Louis, MO, USA) to prevent unspecific binding was added for 30 min. Anti‐CD45 (D9M81; 1 : 200; Cell Signaling Technology, Danvers, MA, USA), 1 : 500 goat‐anti‐rabbit Alexa Fluor 568 as secondary antibody (Thermo Fisher Scientific), and DAPI (1 : 100 00) were used. In a second staining step, melanoma markers were added using directly labeled MCAM (PE) (1 : 100; clone P1H12; EMD Millipore Corp, Burlington, MA, USA) and NG2 (MCSP) (PE) (1 : 50; clone LHM‐2; R&D Systems, Minneapolis, MN, USA). In the last ten cases, two additional directly labeled melanoma markers were added, PMEL17 (SILV) Alexa Fluor 488 (1 : 750; clone HMB45; Novus Biologicals, Littleton, CO, USA) and Melan‐A (MART‐1) Alexa Fluor 488 (1 : 250; clone M2‐7C10; Novus Biologicals), and the CD45 fluorophore conjugate was APC (clone REA747; 1 : 50; Miltenyi Biotec).

### 2.8 Extracellular vesicle isolation

Extracellular vesicles were isolated and prepared for analyses as described in Puhka *et al. *(2017). Plasma samples were diluted with PBS (1 : 2) and filtrated using 0.8 μm Millex AA syringe filters (Merck Millipore, Burlington, MA, USA). EVs were extracted from 0.8 to 1.45 mL of original plasma volume by ultracentrifugation (100 000 ***g***, 2 h, 4 °C) with a TLA‐55 fixed angle rotor. EV was suspended in 100 µl of cold PBS and aliquoted for different downstream analysis. Aliquots were stored in PBS at −80 °C.

### 2.9 Nanoparticle tracking analysis (NTA) and transmission electron microscopy

Nanoparticle tracking analysis (NTA) and electron microscopy (EM) were performed with EVs for quality control. Particle number and size distribution of the EV samples were analyzed with NTA instrument LM14C (NanoSight LTD., London, UK). Videos of 5 × 30 s/sample were recorded at room temperature and analyzed with nanosight NTA 3.0 software (NanoSight LTD.). Statistical testing of differences in particle concentrations was assessed using Wilcoxon signed‐rank test in r after normalization for different starting amounts of plasma. EM was performed with EVs from 0.1 mL of plasma as described in Puhka *et al. *(2017).

### 2.10 MiRNA extraction and sequencing

Total plasma miRNA of 10 melanoma patients and five HDs was extracted from 200 μL plasma from EDTA and Streck tubes collected samples with miRNeasy Serum/Plasma Advanced Kit (Qiagen) according to instructions. The choice of the extraction kit was based on the results of the CANCER‐ID study ‘Multicenter evaluation of circulating plasma miRNA extraction technologies for the development of clinically feasible RT‐qPCR and NGS analytical workflows’. RNA extraction from EVs was performed with miRNeasy Serum/Plasma Kit (Qiagen). QIAseq miRNA libraries were constructed with QIAseq miRNA Library Kit, and Illumina NextSeq High Output sequencing runs were performed (Illumina NextSeq 550) with 5 μL of miRNA. The 3′ adapter sequences and low‐quality bases (Phred score < 10) were trimmed using cutadapt (http://cutadapt.readthedocs.io/en/stable/guide.html), and identical reads were collapsed based on their Unique Molecular Indices (UMIs) sequences. Raw reads below 16 bp and UMI sequences below 10 bp were discarded. The remaining reads were mapped to miRBase using bowtie (http://bowtie-bio.sourceforge.net/index.shtml). The FastQ files are available at geo (accession number http://www.ncbi.nlm.nih.gov/geo/query/acc.cgi?acc=GSE143231).

### 2.11 Differential expression analysis

To assess differential miRNA expression, UMI counts of MM patients were compared to their corresponding HD sample counts. Raw UMI reads of HD and MM samples were therefore normalized together for the groups EDTA plasma, EDTA EV, Streck plasma, and Streck EV. Statistical testing of differences of UMI count medians between EDTA and Streck samples was assessed using Wilcoxon signed‐rank test in r.

To reduce possible bias from the normalization method, normalization was performed using both geNorm (Vandesompele *et al.*, 2002) (GeneGlobe Secondary Data Analysis Tool; Qiagen) and NormFinder (Andersen *et al.*, 2004) (GenEx7; MultiD Analyses AB, Gothenburg, Sweden) algorithms in two separate analyses. Reference genes for the NormFinder algorithm were only considered if their UMI counts exceeded three reads. Subsequent analysis of differential miRNA expression was assessed by *t*‐testing (*P* < 0.05) without correction for multiple testing. A cutoff value of 2 was defined for the fold change (FC) of MM/HD. Only genes with averaged UMI counts> 10 in at least one group were considered in both geNorm and NormFinder analyses.

### 2.12 Orthogonal validation of differentially expressed miRNA

For validation of the results obtained by NGS analysis, 2 μL of miRNA from EDTA and Streck EV samples was reverse transcribed into cDNA using miRCURY LNA RT Kit (Qiagen) according to instructions. miRCURY LNA miRNA PCR assays (Qiagen) of hsa‐miR‐103a‐3p and hsa‐miR‐93‐5p served as reference gene assays, whereas hsa‐miR‐375, hsa‐miR‐215‐5p, and hsa‐miR‐200c‐3p were chosen for orthogonal validation. Reference miRNA were chosen based on geNorm analysis, which identified the 10 most stable genes for NGS normalization, and abundant expression profiles. Assays were performed in triplicates according to the instructions. Analysis was performed with the ΔΔ*C*_q_ method, with Δ*C*_q_ = *C*_q_ [average (hsa‐miR‐103a‐3p + hsa‐miR‐93‐5p)] − *C*_q_ (individual assay of each patient or HD) and ΔΔ*C*_q_ = Δ*C*_q_ (individual patients) − Δ*C*_q_ (average of HD) (Madadi *et al.*, 2019).

## 3 Results

### 3.1 ctDNA mutation analysis

**Fig. 1:**
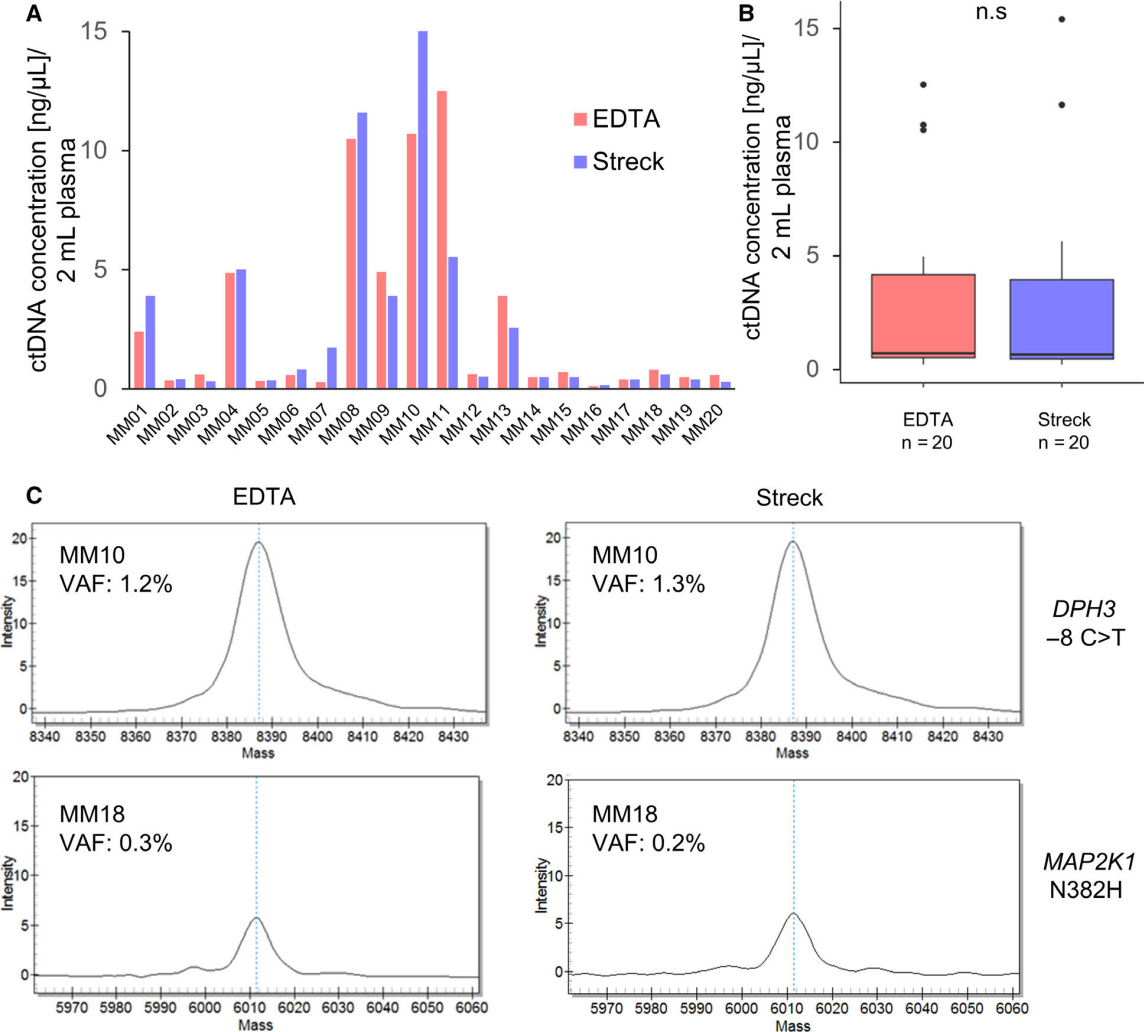
ctDNA concentrations and mutation detection in plasma samples from MM patients. (A) ctDNA concentration (ng·µL^−1^) from 2 mL plasma of individual MM patients in EDTA and Streck tubes. (B) Boxplot depicting the dispersion of ctDNA concentration (ng·µL^−1^) of MM patients (*n* = 20) in EDTA and Streck tubes. No significant difference in ctDNA concentration was found (*P* = 0.96, Wilcoxon signed‐rank test). (C) VAF analysis of patients MM10 and MM18 who showed similar frequencies of somatic mutations for *DPH3* ‐8 C>T and *MAP2K1 N382H* in both EDTA and Streck tubes.

Plasma extraction without substantial hemolysis from blood collected in Transfix tubes was not possible; therefore, with these tubes only CTC analyses could be performed and cfDNA was only isolated from plasma collected in EDTA and Streck tubes. The concentration of the isolated ctDNA ranged from 0.1 to 15 ng·μL^−1^ (Fig. 1A), with no significant difference in ctDNA concentration (mean EDTA: 3.3 ng·μL^−1^ ± 4.6, Streck: 3.0 ng·μL^−1^ ± 4.7) between the two tubes (*P* = 0.96, Wilcoxon signed‐rank test; Fig. 1B).

For the targeted detection of somatic mutations in ctDNA, we used the UltraSEEK® Melanoma Panel covering 86 mutations in 13 key oncogenes (Table S1) implicated in disease progression and therapy response of melanoma in conjunction with the MassARRAY® System (Agena Bioscience, San Diego, CA, USA). A mutation signal produced using this method can be detected down to 0.1% variant allele frequency (VAF) (Mosko *et al.*, 2016).

The VAF in the present study ranged from 0.2% to 2.6%. In total, mutations were detected in 8/12 (66.7%) cases. In all patients except one, the results were identical between the two tubes and the VAF was comparable for each mutation (Table 1, Table S1, Fig. 1C). The most common mutation was *MAP2K1 N382H* (5/12, 42%), followed by *MAP2K1 I111S* (3/12, 25%). *CDKN2A R80X, KIT V559A, BRAF K601E, and NRAS Q61R* were each found in two patients (2/12, 16.7%; Table 1).

**Table 1:** Comparison of VAF of all detected mutations in ctDNA isolated from blood collected in EDTA or Streck tubes.

Pat	Mutation	Nucleotide substitution	VAF %	VAF %
			EDTA	Streck
MM01	CDKN2A R80X	NM_000077.4:c.238C>T	0.3	0.3
	KIT V559A	NM_000222.2:c.1676T>C	0.9	1.1
MM10	BRAF K601E	NM_004333.6:c.1801A>G	0.4	0.5
	DPH3 ‐8C>T	NC_000003.12:g.16306504T>C	1.2	1.3
	RPS27 UTR MUT	NC_000001.11:g.153990763C>T	1.4	1.4
MM13	BRAF K601E	NM_004333.6:c.1801A>G	0.5	WT
	MAP2K1 I111S	NM_002755.3:c.332T>G	WT	0.3
MM14	NRAS Q61R	NM_002524.5:c.182A>G	2.4	2.6
	MAP2K1 N382H	NM_006049.4:c.*162T>G	0.4	0.5
MM15	MAP2K1 I111S	NM_002755.3:c.332T>G	0.5	0.4
	MAP2K1 N382H	NM_006049.4:c.*162T>G	0.6	0.6
	KIT V559A	NM_000222.2:c.1676T>C	0.2	0.3
MM17	MAP2K1 I111S	NM_002755.3:c.332T>G	0.4	0.6
	MAP2K1 N382H	NM_006049.4:c.*162T>G	0.7	0.6
	NRAS Q61R	NM_002524.5:c.182A>G	1.4	1.4
MM18	CDKN2A R80X	NM_000077.4:c.238C>T	0.3	0.2
	MAP2K1 N382H	NM_006049.4:c.*162T>G	0.5	0.6
MM19	MAP2K1 I111S	NM_002755.3:c.332T>G	0.5	0.4
	MAP2K1 N382H	NM_006049.4:c.*162T>G	0.5	0.4

The only exception was patient MM13 in which different mutations could be found in the plasma DNA obtained from EDTA and Streck tubes. In this particular case, there was a *BRAF K601E* mutation found only in the EDTA tube (VAF: 0.7%) and a *MAP2K1 I111S* (VAF: 0.3%) mutation only in the DNA from the Streck tube (Table 1). A second blood draw was not available for this patient to verify these results.

### 3.2 CTC spiking experiments and CTC detection

For CTC enrichment, the ClearCell FX1 System was used. This label‐free CTC enrichment is based on a microfluidic chip that makes use of the different characteristics such as size and inertia of CTC in comparison with normal blood cells (Tan *et al.*, 2016). Spiking experiments were performed to validate the performance of the extraction method combined with melanoma‐specific staining protocols and the influence of plasma removal prior to CTC isolation with the ClearCell FX1 System.

First, the influence of plasma removal on CTC recovery rates was analyzed. Mean recoveries of spiking 50 H1975 GFP‐positive cells into 7.5 mL of EDTA blood (*n* = 4) were 47.0% ± 9.3 without plasma removal. With plasma removal, it was 46.8% ± 12.7 (*P* = 0.85, Wilcoxon signed‐rank test). CTC enrichment from Streck tubes showed a mean recovery of 52% ± 13 after plasma removal. Similar results were obtained when 100 SK‐MEL‐28 cells were used and stained with the first CTC staining protocol for melanoma samples (see 2.7). Here, the recovery rates for EDTA tubes were 49% ± 8.5 and for Streck 44% ± 7.1 when plasma was removed before analysis. Plasma removal does therefore not influence CTC recoveries when using the ClearCell FX1 System. As plasma could not be removed from Transfix tubes without substantial hemolysis, whole blood was analyzed in these tubes.

**Fig. 2:**
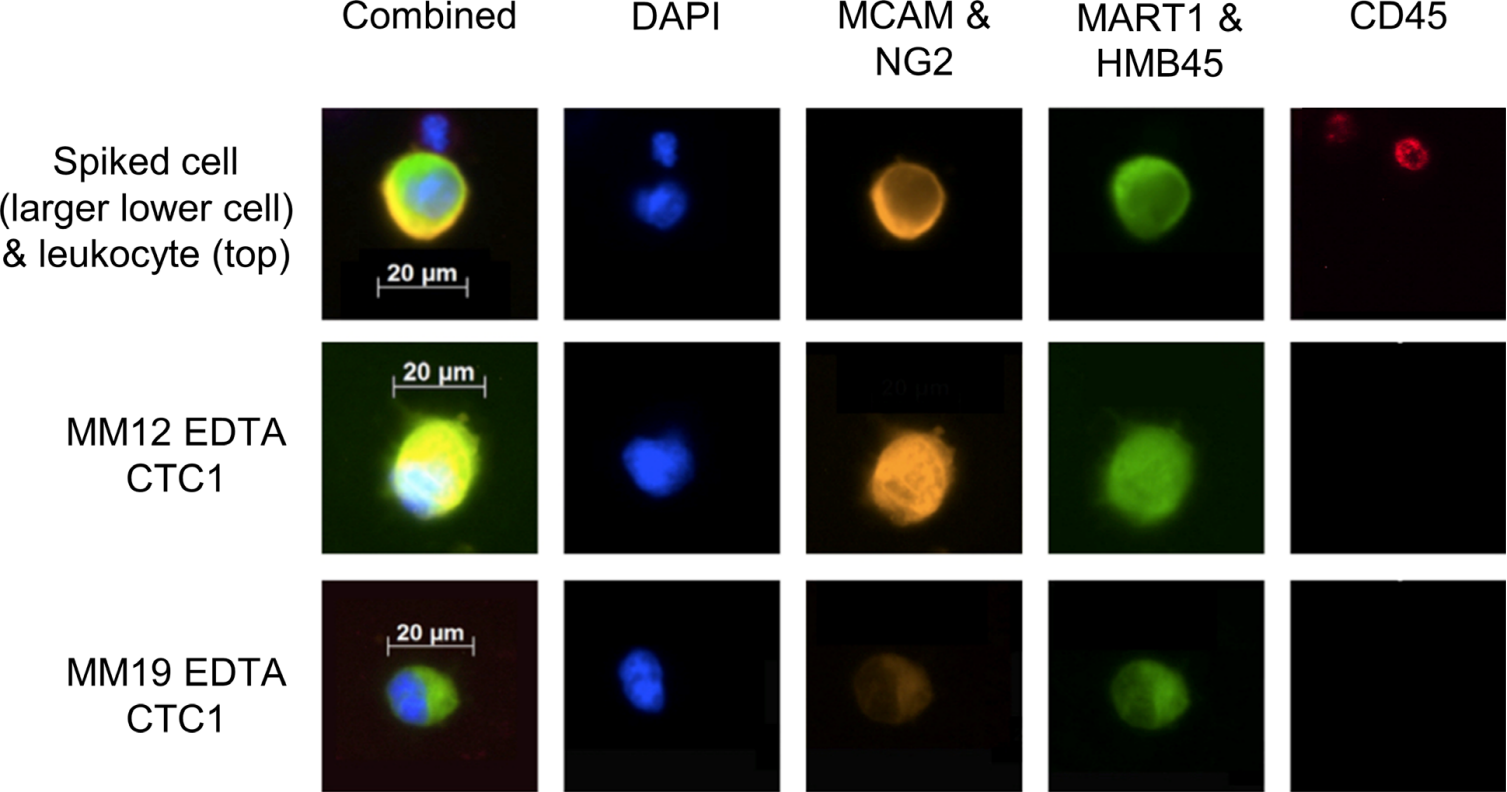
Images of spiked SK‐MEL28 cells in HD blood and CTCs identified in the MM patients using a multicolor immunofluorescence approach. After the isolation with the ClearCell system, the immunofluorescence staining was performed using a combination of four different MM markers (MCAM and NG2 in the PE and MART1 and HMB45 with Alexa 488). As an exclusion marker, CD45 (APC) was used. For nuclear staining, DAPI was used (blue channel). CTCs were positive for the melanoma markers and negative for CD45, while leukocytes were only positive for CD45 and negative for the melanoma markers.

In the patient samples, we found in total CTCs in 15% (3/20) of patients: two CTCs in patient MM01 and one CTC in patient MM12 and MM19 each (Fig. 2). All CTCs were found in the EDTA tube, while none were found in the Streck or Transfix tubes.

### 3.3 Nanoparticle tracking analysis and transmission electron microscopy of EVs

**Fig. 3:**
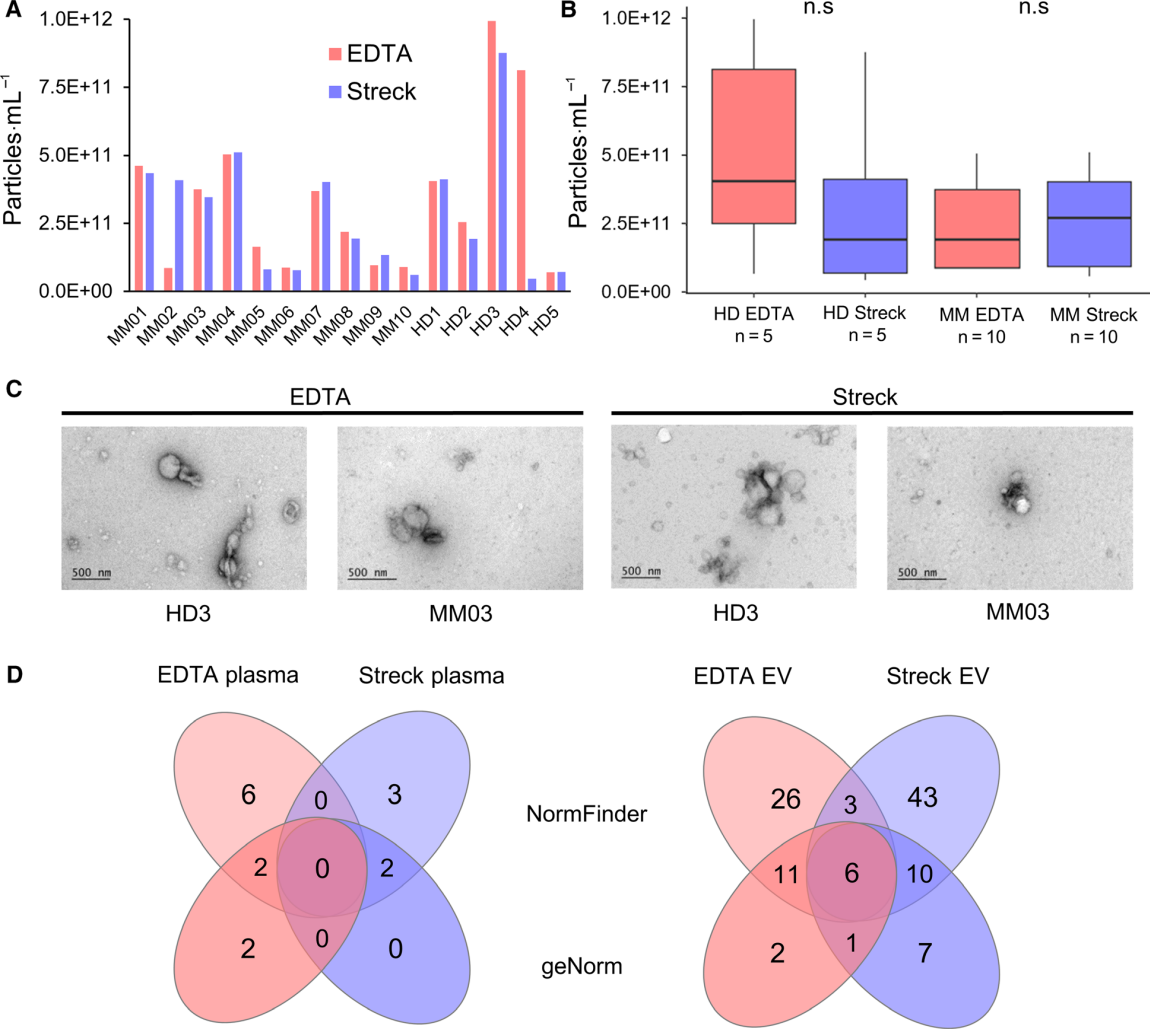
NTA and transmission EM imaging of EVs (A) EV concentration (particles·mL^−1^) of individual MM patients and HDs in EDTA and Streck tubes. (B) Boxplots showing the particle concentration per mL of MM patients (*n* = 10) and HDs (*n* = 5). No significant differences were found between blood collected in EDTA and Streck tubes of HD and MM [*P* = 0.31 (HD), *P* = 0.92 (MM); Wilcoxon signed‐rank test]. (C) EM imaging of EVs isolated from EDTA and Streck tubes from HD3 and MM03 show similarities regarding morphology, size, and sample purity. (D) Venn diagram depicting the number of differentially regulated miRNA from NormFinder and geNorm analyses analyzed in blood collected in either EDTA or Streck tubes.

The mean concentrations of EV particles detected in blood obtained in EDTA tubes were 3.33 × 10^11^ ± 2.77 × 10^11^ and for Streck 2.84 × 10^11^ ± 2.31 × 10^11^ particles·mL^−1^. The obtained particle concentrations were relatively constant between different plasma types from the same donor, but varied between individuals; however, exceptions could be observed in patients MM02 and HD4 (Fig. 3A). The particle concentrations of HD and patients did not differ significantly from each other [*P* = 0.31 (HD), *P* = 0.92 (MM); Wilcoxon signed‐rank test; Fig. 3B].

Similar to particle concentrations, the particle size distributions were relatively constant between different plasma types from the same donor (exceptions MM2 and MM6 and HD1), but showed again more variation between individuals. The particle size distributions of HD and patients did not differ systematically from each other (Fig. S2A). The EV sizes reached up to 1 µm. There was no obvious difference between EDTA vs. Streck plasma nor healthy vs. patient samples regarding EV morphology, sizes, or sample purity (Fig. 3C).

### 3.4 NGS analysis of total plasma miRNA

In order to assess possible differences caused by the collection tube on the quality of downstream analysis by NGS, we first analyzed the raw reads and the UMI counts.

The total raw reads of the averaged individual samples were comparable across all plasma samples and range from approximately 13 to 18 million. The UMI reads, however, were mostly higher in the EDTA than in the Streck samples in both HD and MM fractions, but due to large variance it did not reach significance (paired *t*‐test; Table 2, Fig. S3), with averaged UMI read FC (EDTA/Streck per patient) being 1.8 (range 1.2–2.8, *P* = 0.094) for HDs and 2.6 (range 0.4–4.9, *P* = 0.054) for MM patients.

**Table 2:** miRNA NGS data of total reads of averaged individual HD and MM samples of plasma and EV samples, analyzed in blood collected in either EDTA or Streck tubes.

Read set (average)	EDTA plasma	Streck plasma	EDTA EV	Streck EV
Control				
Total reads	12 918 867	14 473 494	13 627 945	12 763 090
Too short reads	5 230 193	5 869 227	4 405 809	6 672 979
miRNA reads	1 308 356	828 325	7 390 907	3 990 079
miRNA + piRNA UMIs	504 231	288 253	1 399 170	387 321
% UMIs/reads	3.9	2.0	10.3	3.0
Median UMIs	42.5	42.0	5.4	4.0
MM patients				
Total reads	17 902 408	15 079 146	13 323 562	15 583 441
Too short reads	7 157 874	6 205 271	5 231 919	8 248 732
miRNA reads	2 034 688	1 061 176	5 928 317	4 775 402
miRNA + piRNA UMIs	657 612	352 714	1 228 334	615 673
% UMIs/reads	3.7	2.3	9.2	4.0
Median UMIs	45.6	37.0	5.1	4.0

For the analysis of differential miRNA expression in MM compared to HD, two different normalization methods were used. Using the geNorm normalization method, only one miRNA (hsa‐miR‐4799‐3p) was found differentially upregulated and three (hsa‐miR‐205‐5p, hsa‐miR‐4529‐3p, hsa‐miR‐141‐3p) downregulated in the EDTA plasma samples in MM patients compared to HD. In the corresponding Streck plasma samples, one miRNA (hsa‐miR‐506‐3p) was upregulated and one (hsa‐piR‐003731) was downregulated. No overlap between significant miRNA could be detected between the two tubes (Table S2, Fig. 3D). Similar results were obtained using NormFinder as a normalization method. Here, six miRNA were found upregulated and two downregulated in EDTA tubes, while three miRNA were up‐ and two downregulated in Streck tubes (Table S2, Fig. 3D). Both tubes have no miRNA in common, indicating a big influence of the collection tubes on the results.

### 3.5 NGS analysis of EV‐associated miRNA and orthogonal validation of differentially expressed miRNA

Like in the total plasma samples, the averaged raw counts in the EV fractions were similar between different tubes and HD and MM patients and ranged from approximately 13 million (HD EDTA EV) to 15.5 million (MM Streck EV; Table 2, Fig. S3). However, similar to total plasma the UMI reads of the EDTA samples were higher than those of the Streck samples (averaged UMI read FC (EDTA/Streck per patient) of 4.0 (range 0.8–6.8, *P* = 0.059) in HD and 6.5 (range 0.1–18.0, *P* = 0.271) in MM patients).

When analyzing for significantly differentially expressed miRNA in MM‐EVs compared to HD, we identified eight and 11 miRNA that are upregulated in blood from EDTA and Streck tubes, respectively, when using geNorm. Downregulation of 12 miRNA was detected in EDTA samples and of 13 in Streck samples. There was an overlap of seven significant miRNA in the EV samples using geNorm normalization (Fig. 3D, Fig. S2B).

**Fig. 4:**
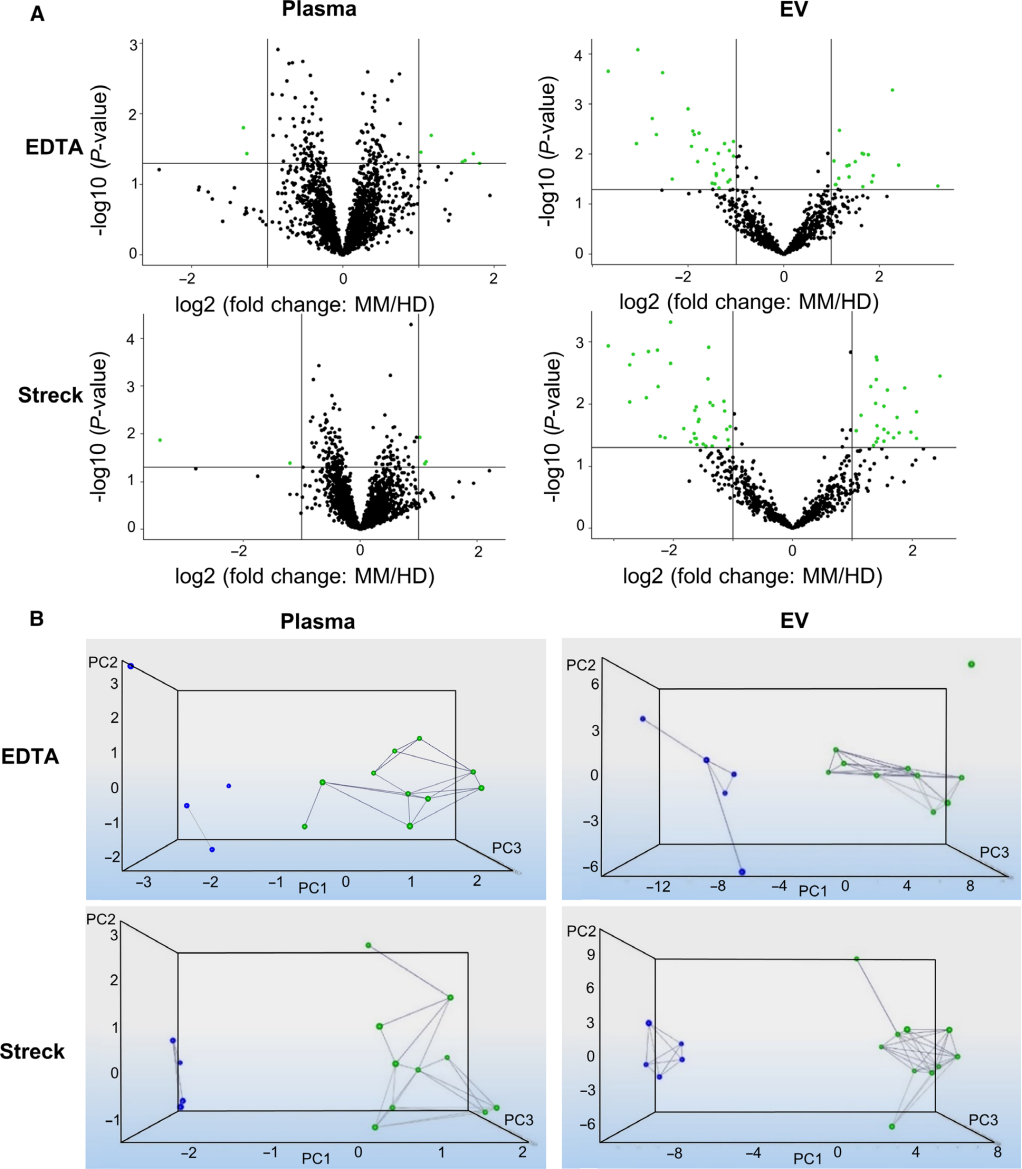
Results of significantly differentially regulated miRNA in MM patients compared to HD (A) After normalization with NormFinder and application of FC (> 2) and *P*‐value (< 0.05) cutoff values, volcano plots illustrate significant differentially expressed genes. Green dots represent statistically significant and differentially regulated miRNA, while miRNA shown as black dots are below these defined thresholds of *P*‐value and FC. In EDTA plasma and EV samples, eight and 46 miRNA were identified with significantly different expression levels, while in Streck plasma and EV samples five and 62 significant miRNA are shown, respectively. (B) Dynamic PCA results after applying thresholds of FC (> 2) and *P*‐value (< 0.05). Blue dots represent HDs; MM samples are shown with a green dot. Since both groups are spatially isolated, a good separation of HDs and MM patients is achieved in both EDTA and Streck samples.

NormFinder results showed slightly more differentially expressed miRNA; 18 miRNA were up‐ and 28 were downregulated in EDTA tubes where 24 were up‐ and 38 downregulated in Streck tubes (Fig. 4A, Table S2). Using NormFinder, nine miRNA were shared between Streck and EDTA. Of these, six significant miRNA were in common with geNorm: hsa‐miR‐375, hsa‐miR‐215‐5p, hsa‐miR‐141‐3p, hsa‐miR‐200a‐3p, hsa‐miR‐200b‐3p, and hsa‐miR‐200c‐3p. Hsa‐miR‐584‐5p was only found significant in geNorm analysis, whereas hsa‐miR‐10a‐5p, hsa‐miR‐10b‐5p, and hsa‐miR‐155‐5p were significantly shared in NormFinder. Notably, none of the identified miRNA was shared between the EV and total plasma fraction.

PCA of these four groups (EDTA plasma, EDTA EV, Streck plasma, and Streck EV) showed a clear separation of the HD samples and the MM patients, indicating that EDTA and Streck tubes were suitable for NGS applications within this setting (Fig. 4B).

To confirm the differential regulation of a subset of the six identified miRNA that were shared by both geNorm and NormFinder analyses, three miRNA (hsa‐miR‐375, hsa‐miR‐215‐5p, and hsa‐miR‐200c‐3p) were chosen due to high FCs and UMI counts in the NGS data. RT‐qPCRs of reversely transcribed miRNA of EDTA and Streck EV samples were performed and normalized against reference miRNA hsa‐miR‐103a‐3p and hsa‐miR‐93‐5p (Table 3). In averaged Streck EV samples, only hsa‐miR‐200c‐3p was found downregulated (1.97‐fold) compared to HD, whereas in EDTA tubes both hsa‐miR‐375 and hsa‐miR‐200c‐3p were downregulated (2.23‐fold and 2.48‐fold, respectively). hsa‐miR‐215‐5p could not be validated in either of the tubes that could be due to the low endogenous level of this miRNA.

**Table 3:** FC values of relative expression of differentially regulated miRNA in HD in comparison with melanoma patients (MM) in either Streck or EDTA EV samples.

	miR‐200c‐3p	miR‐215‐5p	miR‐375
HD Streck/MM Streck	1.97	0.76	1.05
HD EDTA/MM EDTA	2.48	0.95	2.23

## 4 Discussion

In this study of the European CANCER‐ID consortium (http://www.cancer-id.eu), peripheral blood from stage IV melanoma patients was drawn in EDTA, Streck, and Transfix blood collection tubes with the aim to establish a protocol for downstream analysis of ctDNA, CTC, and circulating miRNA from either plasma or EVs from one single blood collection tube. To our knowledge, this is the first study combining all four main liquid biopsy analytes in patients.

Currently, the gold standard for the detection and monitoring of disease progression in melanoma remains tissue biopsy. However, the main sites of melanoma metastases, the brain and lung, can be surgically difficult to access for taking biopsies. Since melanomas harbor an exceptional high tumor heterogeneity and great potential to disseminate to distant organs (Lawrence *et al.*, 2013), being able to establish a multiparameter liquid biopsy approach would be of great advantage for patients (Huang and Hoon, 2016).

Here, ctDNA analysis showed similar concentrations in the blood collected in EDTA and Streck tubes as well as identical mutations when using the highly sensitive MassArray technology (Elazezy and Joosse, 2018). We tested a novel mutation panel consisting of 86 hot‐spot mutations in 13 melanoma relevant genes. In 67% of melanoma patients, a mutation could be detected, indicating that already with this rather restricted panel relevant information can be obtained in most patients. In one patient, a discrepant finding was observed, and thus, in cases with mutations of low VAF a technical replicate run is perhaps advisable.

The analysis of CTCs based on a microfluidic device revealed positive tumor cells in only 15% of patients, and CTCs could only be detected in blood collected in EDTA tubes. The detection of melanoma CTCs has been shown in general to be challenging, mainly due to their large molecular heterogeneity in marker expression. The CTC detection frequency in this study was, however, lower than in most previously published papers on MM patients (Aya‐Bonilla *et al.*, 2017; Koyanagi *et al.*, 2010; Luo *et al.*, 2014; Mocellin *et al.*, 2004). We have recently shown that by using a combined a marker‐dependent (CSPG4 and CD146MACS microbeads) and marker‐independent (Parsortix, Angle plc) detection method, 32% of patients were CTC‐positive with an increase in CTC‐positivity with increased tumor staging (Gorges *et al.*, 2019). However, due to the long processing time of this approach, in this study we decided to test a faster approach enabling the establishment of a clinically relevant multi‐analyte liquid biopsy assay.

Despite the fact that a combination of different markers has shown to lead to a higher number of detected CTCs than the use of a single marker (Freeman *et al.*, 2012; Po *et al.*, 2019), the combination of four markers (MCAM, NG2, MART‐1, and HMB45) did not lead to an increase in CTC detection compared to only MCAM and NG2 in our study. Interestingly, there is also evidence that the markers on CTCs do not always correlate with the markers expressed on the primary tumor, which leads to the conclusion that CTCs could derive from a rare subpopulation of tumor cells (Aya‐Bonilla *et al.*, 2019; Gorges *et al.*, 2019; Gray *et al.*, 2015), making the choice of right detection marker even harder. Therefore, due to the obviously large heterogeneity of melanoma CTCs and their marker expression, it is important to establish unbiased and marker‐independent detection methods and to focus on physical qualities such as cell size, morphology, and/or rigidity (Marsavela *et al.*, 2018). Therefore, methods such as the ClearCell device could be the right choice of enrichment but would need to be combined with an optimized staining protocol or other detection methods such as mRNA or mutation/CNA detection.

Extracellular vesicles have attracted increasing attention as liquid biopsy markers that also influence tumor biology (Anfossi *et al.*, 2018; Cheng *et al.*, 2014; Hoshino *et al.*, 2015). Here, we tested an NGS‐based miRNA detection and quantification workflow for both total plasma miRNA and EV‐associated miRNA. This is, to the best of our knowledge, the first unbiased pre‐analytical liquid biopsy study investigating the melanoma miRnome of whole plasma and EVs by NGS. EV concentrations and size distributions as well as morphology, size, and sample purity did not differ significantly between EDTA and Streck tubes. The NGS results using Streck tubes showed fewer UMI counts compared to EDTA tubes especially in EVs. In EDTA samples, on average 4.0 (HD) and 6.5 (MM) times more UMIs were detected compared to Streck samples, indicating that the sensitivity of miRNA detection is diminished in Streck tubes compared to EDTA tubes.

Surprisingly, more significantly differentially expressed miRNA were detected in the EV fraction compared to the total plasma. Furthermore, differential miRNA expression analysis revealed no overlap between EDTA and Streck tubes in the total plasma samples, whereas an overlap of six miRNA in EV samples between the tubes was found. Therefore, in plasma the total miRNome stability seems to be more heavily influenced by the blood collection tube than in the EVs‐associated miRNome. Differential expression of hsa‐miR‐200c‐3p and hsa‐miR‐375 could be confirmed in an orthogonal validation approach by qPCR in EV samples. Downregulation of hsa‐miR‐215‐5p in patients, however, could not be verified. These EV results are validated by the fact that we detected miRNA changes relevant in MM development and progression such as deregulation of miR‐141‐3p and the miR‐200 family (Feng *et al.*, 2014; van Kempen *et al.*, 2012; Verrando *et al.*, 2016; Xu *et al.*, 2012).

Since differences in library size or sequencing depth can influence the differential expression analysis, appropriate normalization of the data is indispensable (Dillies *et al.*, 2013). In order to avoid bias from normalization methods, we analyzed the samples using two different normalization algorithms: geNorm and NormFinder. Although significant results were obtained with both algorithms, only a small number of differentially regulated miRNA were overlapping, indicating that miRNA analyses are dependent on both normalization method and choice of blood collection tubes. Overall, it is important to note that instead of identifying novel melanoma markers here, the main aim of this study was to identify a workflow for multiparameter liquid biopsy assessment. In order to find new markers, a much larger study cohort would be needed, as well as the confirmation through an independent data set.

## 5 Conclusion

The present study indicates that the described NGS workflow is well suited for miRNA identification and that both EDTA and Streck tubes are suitable for this analysis. Although in both total and EV plasma fractions fewer NGS reads of miRNA could be obtained in Streck tubes, significant results could nevertheless be obtained. Therefore, miRNA results obtained largely depend on the blood collection tubes and normalization method. Interestingly, circulating miRNA found in the total plasma or EVs are different, which can be explained by the fact that total plasma contains circulating EV‐free miRNA bound to proteins in addition to EVs, indicating a clinical benefit of assays of both compartments.

If samples can be processed rapidly after blood draw, EDTA tubes show a superior efficiency of CTC detection and are well suited for evaluation of all three analytes. ctDNA quantity and quality as well as VAF analysis, however, remain largely unaffected by the choice of tube if processed immediately. Nonetheless, in a clinical setting quick processing is not always possible; therefore, Streck tubes represent a good alternative as these tubes have been shown to conserve ctDNA, CTCs, and miRNA over longer time periods compared to EDTA tubes (Alidousty *et al.*, 2017; Diaz *et al.*, 2016; Qin *et al.*, 2014; Ward Gahlawat *et al.*, 2019). Taken together, ctDNA, CTC, and miRNA can be analyzed from a single blood collection tube, but the choice of the tube affects the outcome of the analysis, underlining the importance of pre‐analytical factors.

## Conflict of interest

Mikael Kubista is an employee of TATAA Biocenter AB. Alexander Sartori and Darryl Irwin are employees of Agena Bioscience GmbH. Elina Serkkola and Taija af Hällström are employees of Orion Pharma. Markus Sprenger‐Haussels, Melanie Hussong, and Jonathan Shaffer are employees of QIAGEN Inc/GmbH.

## Author contribution

SS, ES, TaH, MS‐H, EL, KP, and HW performed the study concept and design of the study (complete Cancer‐ID team). SS, LL, MK, MH, AS, JS, DI, and HW analyzed the data. JS, L‐BS, JS, CG, SWS involved in the patient sample collection. SS, LL, KB, RG, BV, MH, and JH performed experiments. SS, LL, EL, MS‐H, JS, and HW involved in the data interpretation. SS, LL, KP, and HW drafted the manuscript. All authors approved final manuscript.

## Supporting information


**Fig. S1.** Overview of the experimental procedure. Blood (7.5 mL) was collected from 20 melanoma patients and five healthy in EDTA, Streck and Transfix tubes and PBMC fraction was separated from plasma by centrifugation (500 ***g***, 10 min). CTC isolation from the PBMC fraction was performed by the ClearCell device (Biolidics Limited). CTCs were identified by immunofluorescence staining. After further centrifugation of the plasma fraction (EDTA: 2500 ***g***, 10 min; Streck: 6000 ***g***, 10 min), ctDNA was extracted with QIAamp MinElute ccfDNA Midi Kit (QIAGEN) from Streck and EDTA plasma samples (1.5–2 mL) before analyzing 86 hot‐spot mutations in 13 genes by the UltraSEEK chemistry (Agena Bioscience). After two additional centrifugation steps of the plasma fraction (2500 ***g***, 10 min and 2500 ***g*** for 15) miRNAs were extracted with miRNeasy Serum/Plasma Advanced Kit and miRNeasy Serum/Plasma Kit (QIAGEN) from total plasma and from EVs, respectively. EVs were isolated by ultracentrifugation. QIAseq miRNA libraries were produced and sequenced (Illumina NextSeq 550). The reads were mapped to miRBase and identical reads were collapsed based on their UMIs sequences. The data was normalized using NormFinder and geNorm. *ctDNA concentration measured from *n* = 20; mutation analysis assessed from *n* = 12.

**Fig. S2.** Particle size distributions of EVs and geNorm results of significantly differentially regulated miRNAs in MM patients compared to HD. (A) Particle size distributions of HD and patients, assessed by NTA. (B) After normalization with geNorm and application of fold‐change (> 2) and *P*‐value (< 0.05) cutoff values, volcano plots illustrate significant differentially expressed genes. Green dots represent statistically significant and differentially regulated miRNAs while miRNAs shown as black dots are below these defined thresholds of *P*‐value and FC.

**Fig. S3.** Analysis of UMIs. Density distributions of raw averaged (left) and exemplary single (right) HD UMI counts in plasma and EV samples, analyzed in blood collected in either EDTA (red) or Streck (blue) tubes.

**Table S1.** Detailed summary of the UltraSEEK™ Melanoma Panel The panel (Agena Bioscience) covers 86 hot‐spot mutations in 13 genes including nucleotide change, amino acid change and COSMIC ID.

**Table S2.** Detailed summary of differentially regulated miRNAs Table of miRNAs differentially regulated in plasma and EV samples, analyzed in blood collected in either EDTA or Streck tubes using normalization algorithms NormFinder or geNorm and applying fold‐change (> 2) and *P*‐value (< 0.05) cut‐off values.
