# Supplementary figures and images for: Pre‐analytical factors affecting the establishment of a single tube assay for multiparameter liquid biopsy detection in melanoma patients
Source: Mol Oncol. 2020 Apr 4;14(5):1001–15. doi: 10.1002/1878-0261.12669 (PMC7191195; doi:10.1002/1878-0261.12669)

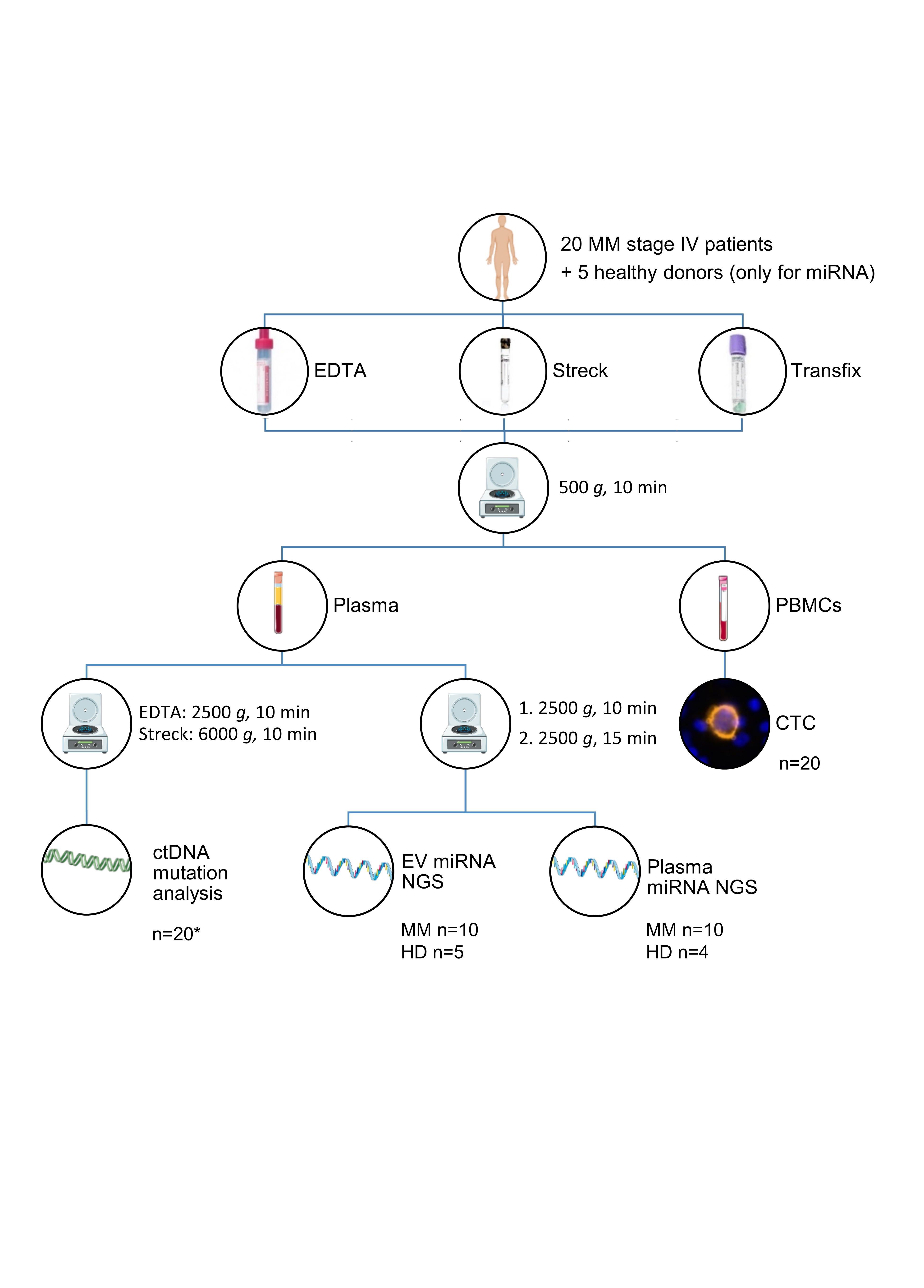

Supplement: Supplementary file 1 — Fig. S1. Overview of the experimental procedure. Blood (7.5 mL) was collected from 20 melanoma patients and five healthy in EDTA, Streck and Transfix tubes and PBMC fraction was separated from plasma by centrifugation (500 g, 10 min). CTC isolation from the PBMC fraction was performed by the ClearCell device (Biolidics Limited). CTCs were identified by immunofluorescence staining. After further centrifugation of the plasma fraction (EDTA: 2500 g, 10 min; Streck: 6000 g, 10 min), ctDNA was extracted with QIAamp MinElute ccfDNA Midi Kit (QIAGEN) from Streck and EDTA plasma samples (1.5–2 mL) before analyzing 86 hot‐spot mutations in 13 genes by the UltraSEEK chemistry (Agena Bioscience). After two additional centrifugation steps of the plasma fraction (2500 g, 10 min and 2500 g for 15) miRNAs were extracted with miRNeasy Serum/Plasma Advanced Kit and miRNeasy Serum/Plasma Kit (QIAGEN) from total plasma and from EVs, respectively. EVs were isolated by ultracentrifugation. QIAseq miRNA libraries were produced and sequenced (Illumina NextSeq 550). The reads were mapped to miRBase and identical reads were collapsed based on their UMIs sequences. The data was normalized using NormFinder and geNorm. *ctDNA concentration measured from n = 20; mutation analysis assessed from n = 12. [file MOL2-14-1001-s001.tif]

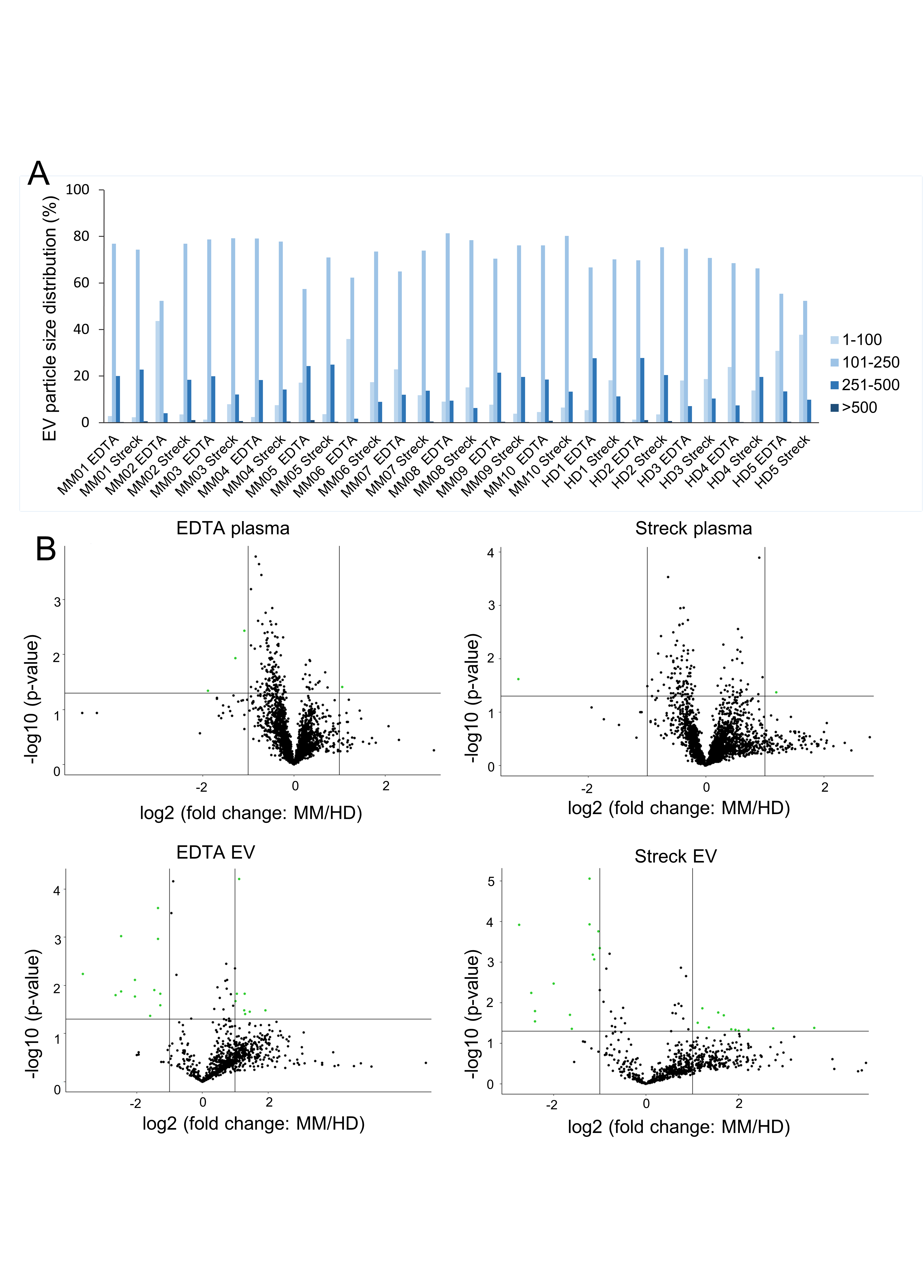

Supplement: Supplementary file 2 — Fig. S2. Particle size distributions of EVs and geNorm results of significantly differentially regulated miRNAs in MM patients compared to HD. (A) Particle size distributions of HD and patients, assessed by NTA. (B) After normalization with geNorm and application of fold‐change (> 2) and P‐value (< 0.05) cutoff values, volcano plots illustrate significant differentially expressed genes. Green dots represent statistically significant and differentially regulated miRNAs while miRNAs shown as black dots are below these defined thresholds of P‐value and FC. [file MOL2-14-1001-s002.tif]

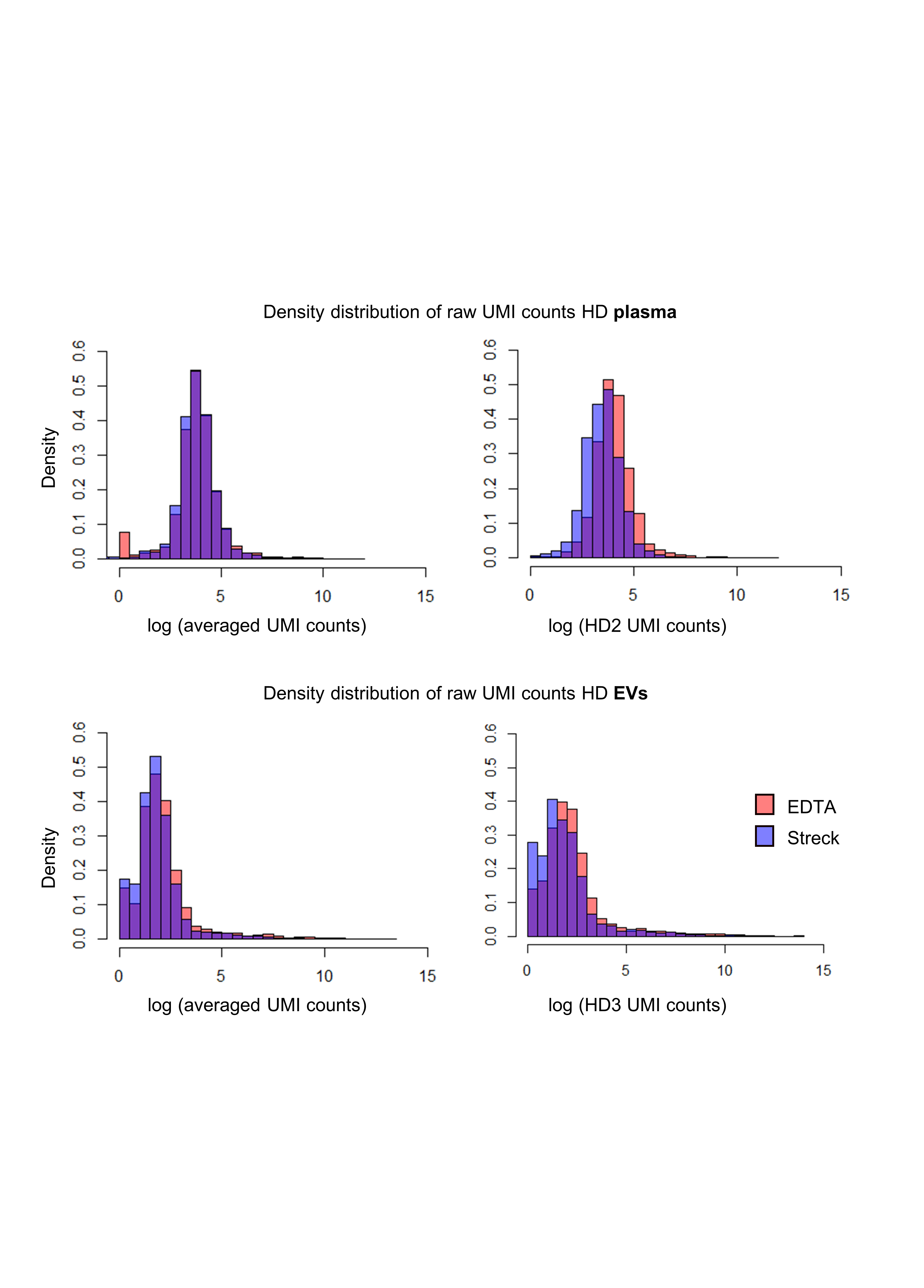

Supplement: Supplementary file 3 — Fig. S3. Analysis of UMIs. Density distributions of raw averaged (left) and exemplary single (right) HD UMI counts in plasma and EV samples, analyzed in blood collected in either EDTA (red) or Streck (blue) tubes. [file MOL2-14-1001-s003.tif]
